# Ultrasonography as a sensitive and specific diagnostic modality for the detection of ectopic ureters in urinary incontinent dogs

**DOI:** 10.1111/vru.13055

**Published:** 2022-01-22

**Authors:** Oliver Taylor, Rebekah Knight, Marie‐Aude Genain, Laura Owen

**Affiliations:** ^1^ Queen Mother Hospital for Animals Royal Veterinary College Hatfield UK; ^2^ Department of Veterinary Medicine Queen's Veterinary School Hospital Cambridge UK

**Keywords:** canine, ultrasound, ureteral ectopia, urinary incontinence

## Abstract

Ultrasonography is a widely available diagnostic modality for the identification of dogs with suspected ureteral ectopia; however published studies detailing its sensitivity and specificity are currently lacking. The aim of this retrospective, descriptive, diagnostic accuracy study was to evaluate the sensitivity and specificity of ultrasonography for the diagnosis of ureteral ectopia in incontinent dogs presenting to a referral institution, using cystoscopy as the gold standard. Medical records of urinary incontinent dogs presenting to a single institution (n = 38) were retrospectively reviewed for the presence of ureteric insertion abnormalities and concurrent urinary tract abnormalities. Ultrasonographic findings were compared with those from cystoscopic examination to determine diagnostic accuracy. The relationship between the presence of concurrent urinary tract abnormalities and ureteral ectopia was assessed using an independent samples *t*‐test and Mann–Whitney test. Statistical significance was set at *P *≤ 0.05. Ultrasonography had a sensitivity of 93.5%, specificity of 100%, and diagnostic accuracy of 95% when identifying dogs with ureteral ectopia. When classifying individual ureters as ectopic or non‐ectopic, sensitivity was 87.8% and specificity was 86.7%. Dogs with ureteral ectopia had significantly more concurrent urinary tract abnormalities on ultrasound than unaffected dogs (*P* = 0.004). Ectopic ureters were associated with significantly more concurrent ipsilateral upper urinary tract ultrasonographic abnormalities than unaffected ureters (*P* < 0.001). Ultrasonography performed by an experienced ultrasonographer is a sensitive and specific screening tool for canine ureteral ectopia, which eliminates the need for heavy sedation, general anesthesia, and advanced imaging, although it should not be relied upon as the sole diagnostic modality for the assessment of individual ureters.

**Abbreviations**
AUS: abdominal ultrasonography
CUTA: concurrent urinary tract abnormalities
CIUTA: concurrent ipsilateral urinary tract abnormalities
ECVDI: European College of Veterinary Diagnostic and Imaging
ECVS: European College of Veterinary Surgery
EU: ectopic ureters
UEU: upper urinary tract (kidney/ureter) associated with an ectopic ureter
Non‐UEU: upper urinary tract (kidney/ureter) associated with a non‐ectopic ureter.

## 1 INTRODUCTION

Ureteral ectopia is the most common cause of urinary incontinence in juvenile dogs.^1^ It is a congenital abnormality in which one or both ureteral orifices are inappropriately located distal to the bladder trigone. Intramural and extramural ectopic ureters (EUs) have been reported in dogs, with >95% of cases being intramural.^2^, ^3^, ^4^ Female dogs are often more severely urinary incontinent and are over‐represented in dogs presenting with urinary incontinence secondary to ureteral ectopia, which has been theorized to be due to their shorter urethral length.^3^, ^5^ It is currently hypothesized that there is a genetic basis for this condition with Labrador Retriever, Golden Retriever, Newfoundland, Siberian Husky , Poodle, soft‐coated Wheaten Terrier, West Highland White Terrier, and Skye Terrier breeds being predisposed.^1^, ^3^, ^5^, ^6^ Ureteral ectopia is usually suspected based on clinical history, signalment, and physical examination findings. Diagnostic imaging is then utilized to evaluate the path of the distal ureter, identify the ureteral orifice, and to identify concurrent abnormalities such as hydroureter and hydronephrosis.

Historically, radiographic excretory urography combined with retrograde urethrocystography has been relied upon to identify EUs or associated pathology.^2^, ^7^, ^8^, ^9^ Excretory urography has been reported to identify the location of ectopic ureteral orifices correctly in 66–78.2% of cases,^2^, ^7^, ^8^ with fluoroscopy and evacuation of rectal contents shown to improve sensitivity.^10^ More recently, CT excretory urography (CT) and four‐dimensional CT excretory urography (4D‐CTEU) have been used for the diagnosis of EUs with a reported 73–100% and 97% sensitivity, respectively.^10^, ^11^, ^12^ With the advancement of endoscopic technology, cystoscopy has been described as the gold standard for EU diagnosis as it allows the operator to visualize the ureteral orifices directly, with a reported sensitivity of 100%, as well as enabling concurrent laser ablation of intramural EUs.^5^, ^8^, ^10^ One of the limitations of excretory urography, CT, and cystoscopy is the requirement for general anesthesia or heavy sedation. Intravenous administration of iodine‐based contrast medium for excretory urography and CT is associated with a low risk of adverse reactions and acute kidney injury.^13^ Abdominal ultrasonography (AUS) can provide a detailed assessment of the upper urinary tract and the position of the ureters, without the need for general anesthesia (and in some cases without sedation) or intravenous contrast injection.^5^ While its use has been recommended alongside cystoscopy, there is limited information on the sensitivity or specificity of this imaging modality in dogs with ureteral ectopia. Lamb et al reported a sensitivity of 91% for the identification of EUs by AUS when compared with contrast radiographs or surgical and necropsy findings in 14 dogs.^14^ To the best of the authors’ knowledge, there are no published studies evaluating the diagnostic accuracy of ultrasound for ureteral ectopia using cystoscopy as the gold standard for comparison to date.

The primary aim of the current study was to evaluate the sensitivity and specificity of AUS for diagnosis of ureteral ectopia in dogs with urinary incontinence presenting to a referral institution, using cystoscopic findings as the gold standard for comparison. We hypothesized that AUS would have a sensitivity >85% for a diagnosis of EU when compared to cystoscopy and therefore would be a useful imaging modality in clinical cases. The secondary aim was to assess whether any other AUS findings could be used to aid the diagnosis of ureteral ectopia.

## 2 MATERIALS AND METHODS

### 2.1 Selection and description of subjects

Medical records of dogs from a single institution (Queen's Veterinary School Hospital), presenting for investigation of urinary incontinence between December 2014 and February 2020 were reviewed for this retrospective, diagnostic accuracy, descriptive study. Data collection was approved by the institution's Ethics and Welfare Committee prior to study initiation. The inclusion criteria were the use of both ultrasonography and cystoscopy during the diagnostic investigation within the same diagnostic process (allowing for a maximum of 3 months between both diagnostic techniques). Cases were excluded from the study if other imaging modalities had been performed alongside AUS and cystoscopy, as this may have affected the interpretation of the AUS findings. Dogs that had previously had urinary tract surgery were excluded from the study. Final decisions for inclusion or exclusion were made by a European College of Veterinary Surgery (ECVS)‐certified veterinary surgeon (L.O.).

### 2.2 Data recording and analysis

Medical records were reviewed by a small animal veterinary intern (O.T.). Recorded data included the following: signalment, presenting complaint(s), AUS findings, and cystoscopy findings. Reports of AUS performed by a European College of Veterinary Diagnostic and Imaging (ECVDI)‐certified veterinary radiologist, or a radiology resident under the supervision of an ECVDI‐certified veterinary radiologist, were reviewed and urinary tract abnormalities were recorded (Tables 1 and 2). The presence of ureteral ectopia was documented. Within the AUS report, each ureter was classified as either intramural ectopic, extramural ectopic, or normal. The dog was then categorized as either “positive for ureteral ectopia” or “negative for ureteral ectopia” based on AUS. All concurrent urinary tract abnormalities (CUTA) included within the radiologist's report were recorded. The CUTA specifically affecting the ipsilateral kidney or ureter were subclassified as concurrent ipsilateral upper urinary tract abnormalities (CIUTA).

**TABLE 1:** A table summarising the frequency of concurrent urinary tract abnormalities (CUTA) in the ectopic ureter (EU) and non‐ectopic ureter (non‐EU) patient populations

Ultrasonographic Observed Abnormalities	Observations in EU population (n = 31)	Frequency in EU population (n = 31)	Observations in non‐EU population (n = 7)	Frequency in non‐EU population (n = 7)
Intra‐pelvic Bladder	6	19%	2	29%
Indistinct Bladder Neck	4	13%	0	0%
Hyperechoic Material in Bladder	3	10%	1	14%
Short/Wide Urethra	2	6%	0	0%
Ureteral Dilation	21	68%	0	0%
Renal Pelvis Dilation	23	74%	3	43%
Reduced Cortico‐medullary Definition	7	23%	0	0%
Renal Medullary Cyst	3	10%	0	0%
Ureterocoele	1	3%	0	0%
Pyelonephritis	1	3%	0	0%
Renal Dysplasia	1	3%	0	0%
Irregular Renal Contour	1	3%	0	0%

**TABLE 2:** A table summarizing the frequency of concurrent ipsilateral urinary tract abnormalities (CIUTA) observed in upper urinary tracts (kidney/ureter) associated with an ectopic ureter (UEU) and upper urinary tracts (kidney/ureter) associated with a non‐ectopic ureter (non‐UEU) study populations. Two ureters that could not be identified as either the left or right ureter have been excluded from the above table's population

Ultrasonographic Observed Abnormalities	Observations in UEU population (n = 42)	Frequency in UEU population (n = 42)	Observations in non‐UEU population (n = 32)	Frequency in non‐UEU population (n = 32)
Renal Pelvis Dilation	28	67%	8	25%
Ureteral dilation	25	60%	1	3%
Reduced Cortico‐medullary Definition	9	21%	1	3%
Renal Medullary Cyst	3	7%	0	0%
Ureterocoele	1	2%	0	0%
Pyelonephritis	1	2%	0	0%
Renal Dysplasia	1	2%	0	0%
Irregular Renal Contour	1	2%	0	0%

Ureteral and renal pelvis diameters included within the radiologist report were recorded. The reports from the cystoscopic examination performed by a board‐certified surgeon for each dog were reviewed and each ureter was classified as either ectopic or non‐ectopic. The dog was then categorized as either “positive for ureteral ectopia” or “negative for ureteral ectopia.”

**FIGURE 1:**
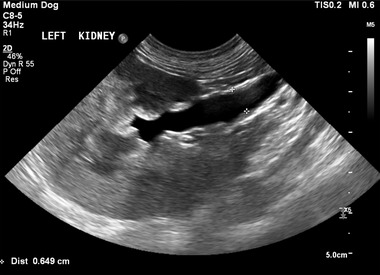
Transverse ultrasonographic image of the left kidney of a dog with an ectopic left ureter acquired with the patient in right lateral recumbency using a Phillips EPIQ 7 ultrasonography machine with a curvilinear 8–5 MHz probe (Philips UK Ltd, Guildford, UK). The kidney shows poor to absent corticomedullary differentiation and an irregularly dilated renal pelvis. The ureter is dilated to 0.6 cm

For cases with incomplete medical records, AUS images were retrieved and measurements were performed by a small animal veterinary intern (O.T.), in consultation with an ECVDI‐certified veterinary radiologist (M.A.G.). The still transverse image of the kidney and ureters were evaluated using commercially available software (RadiAnt DICOM Viewer 2020.1 64‐bit). To determine the renal pelvis diameter, measurements were taken from still transverse images of the central level of the pelvis. If the renal pelvis diameter was greater than 3 mm, the renal pelvis was deemed to be dilated.^15^ To determine the ureteral diameter, measurements were taken from a longitudinal view at a central point of the ureter (Figure 1). Ureteral peristalsis was accounted for by only including the maximal ureteral diameter. The ureter was defined as dilated if the maximum diameter was greater than 4 mm.^16^

### 2.3 Statistics

All statistical analysis was performed by a small animal veterinary intern (O.T.), in consultation with an ECVS‐certified veterinary surgeon (L.O.), using commercially available statistics software (IBM SPSS Statistics Software, version 26; SPSS). The distribution of data for continuous variables was assessed for normality by visual inspection of their histograms. Descriptive statistics were performed, with results expressed as frequencies (categorical variables), mean ± standard deviation (parametric continuous variables), or median and range (non‐parametric continuous variables). The sensitivity, specificity, predictive values, and overall diagnostic accuracy of AUS for correct classification of each individual ureter as ectopic or normal, and for an overall diagnosis of ureteral ectopia in each dog were calculated. The mean number of overall CUTA in dogs with and without EUs were compared using an independent samples *t*‐test and the cut‐off values for predicting a diagnosis of EUs were suggested based on the calculation of sensitivity and specificity at each level. The median number of CIUTA in EUs compared with non‐EUs were compared using a Mann–Whitney test and the cut‐off values for predicting a diagnosis of EUs were suggested based on the calculation of sensitivity and specificity at each level. Statistical significance was set at *P* < 0.05.

## 3 RESULTS

Forty‐six dogs were referred for investigation of urinary incontinence at the Queen's Veterinary School Hospital (QVSH) between December 2014 and February 2020. All underwent AUS and cystoscopy as part of the diagnostic investigation. Six dogs were excluded from the study as radiography with intravenous excretory urography had been performed at the same time as AUS. Two dogs were excluded from the study as ureteroneocystostomy had been performed prior to referral.

A total of 38 dogs (76 ureters) were included in analyses for the current study. This study population comprised 31 entire females (81%), six neutered females (16%) and one entire male (3%). The median age at the time of presentation was 5 months (range 1.25–132 months). Twenty‐eight of 38 (74%) dogs were juvenile and 10 of 38 dogs (26%) were adult at the time of presentation. Sixty‐three percent (24/38) were Golden Retrievers, 11% (4/38) Labradors, 5% (2/38) mixed breeds, 5% (2/38) French Bulldogs, and one dog each of Boxer, Shetland Sheepdog, Miniature Dachshund, Border Terrier, Hungarian Vizsla, and Welsh Springer Spaniel.

**FIGURE 2:**
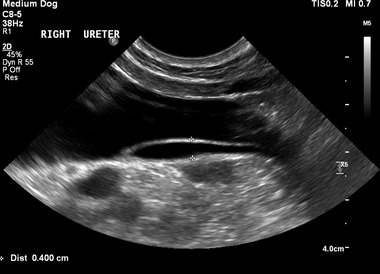
Sagittal ultrasonographic image of the urinary bladder of a dog with a right intramural ectopic ureter acquired with the patient in left lateral recumbency using a Phillips EPIQ 7 ultrasonography machine with a curvilinear 8–5 MHz probe (Philips UK Ltd, Guildford, UK). The ectopic ureter (between calipers) can be seen as a tubular structure encroaching into the bladder lumen and following the bladder wall caudally

In all dogs, the AUS was performed or supervised by one of two board‐certified veterinary radiologists (MAG), with the dog conscious or lightly sedated depending on its temperament. The ultrasonographic examination was performed using one of three machines (Phillips EPIQ 7 with a curvilinear 8–5 MHz or linear 18–5 MHz probe, Philips UK Ltd, Guildford, UK; Philips HDI 5000 SonoCT with a curvilinear 8–5 MHz probe, Philips UK Ltd, Guildford, UK; and an Esaote Mylab 8 exp with a curvilinear 8–5 MHz probe, Esaote Ltd, Genoa, Italy). Using Doppler and brightness mode ultrasound, the location of the ureteral openings was assessed. If the ureteral openings were not readily identifiable, furosemide (Furosemide (Dimazon; MSD Animal Health UK, 1 mg/kg) was administered intravenously to aid identification of the ureteral jets by increasing urine production and reducing the specific gravity of the urine to give greater contrast. Ultrasonographic diagnosis of an ectopic ureter was based on the presence of several characteristics. These included the lack of a ureteral jet identified within the bladder using Doppler, visualization of a tubular structure entering the urinary tract distal to the bladder neck, visualization of the papilla distal to the bladder neck, or visualization of a jet of urine distal to the bladder neck using color Doppler. Differentiation between intramural and extramural ectopic ureters was reliant on the visualization of the distal ureteral pathway. Intramural ectopic ureters were identified by visualization of the distal ureter implanting into the bladder wall at the level of the trigone, with the visualization of a tubular structure traveling parallel to it to a point distal to the bladder neck (Figure 2). Extramural ectopic ureters were identified by visualizing using Doppler, a ureteral jet distal to the bladder neck with the lack of a tubular structure within the bladder wall proximal to this point or by visualizing the ureter bypassing the bladder and implanting distal to the bladder trigone.

**FIGURE 3:**
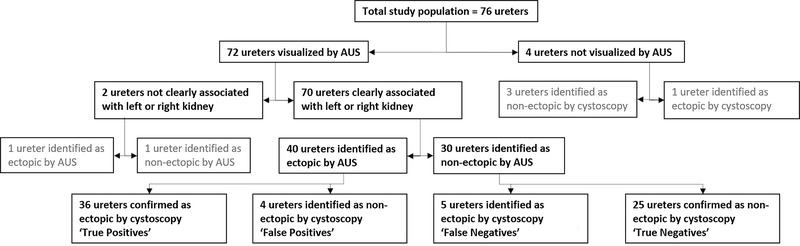
Flow chart illustrating the diagnostic accuracy of abdominal ultrasonography (AUS) when utilized to determine whether an individual ureter was ectopic or non‐ectopic. The ureters demarcated by grey text have been excluded from the sensitivity and specificity calculations for the diagnosis of EU by AUS for individual ureters

On AUS examination, 92% (70/76) of ureters were identified as the left or right ureter and defined as either ectopic or non‐ectopic (Figure 3). Four of 76 (5%) ureters could not be identified by ultrasonography. For two of 76 (3%) ureters, it was not possible to determine whether the visualized ureter was originating from the left or right kidney. Forty of 70 (57%) ureters were identified to be ectopic and 30 of 70 (43%) ureters were described as non‐ectopic. Thirty‐nine of 40 (98%) ureters identified as ectopic were described as intramural.

**FIGURE 4:**
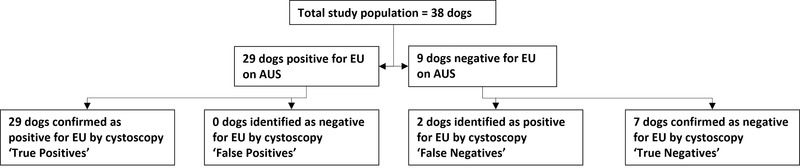
Flow chart illustrating the diagnostic accuracy of abdominal ultrasonography when utilized to determine whether a patient was positive or negative for ureteral ectopia

Examination with AUS identified both ureters in 34 of 38 dogs (89%). Eleven of 34 dogs (32%) were suspected to have bilateral intramural EUs, one (3%) dog was reported to have bilateral EUs with one intramural and one extramural ureter, 13 dogs (38%) had unilateral intramural ureteral ectopia identified and nine dogs (26%) had both ureters identified as non‐ectopic. AUS identified only one ureter in four dogs; three of these four dogs had evidence of at least one ectopic ureter and were subsequently classified as being positive for ureteral ectopia. Twenty‐nine of 38 dogs (76%) were diagnosed with ureteral ectopia (unilateral or bilateral) based on AUS examination (Figure 4). In one dog (3%), unilateral intramural ureteral ectopia was described but the affected ureter could not be identified as either the left or right ureter and in this dog, there were no CIUTA to assist in this identification.

**FIGURE 5:**
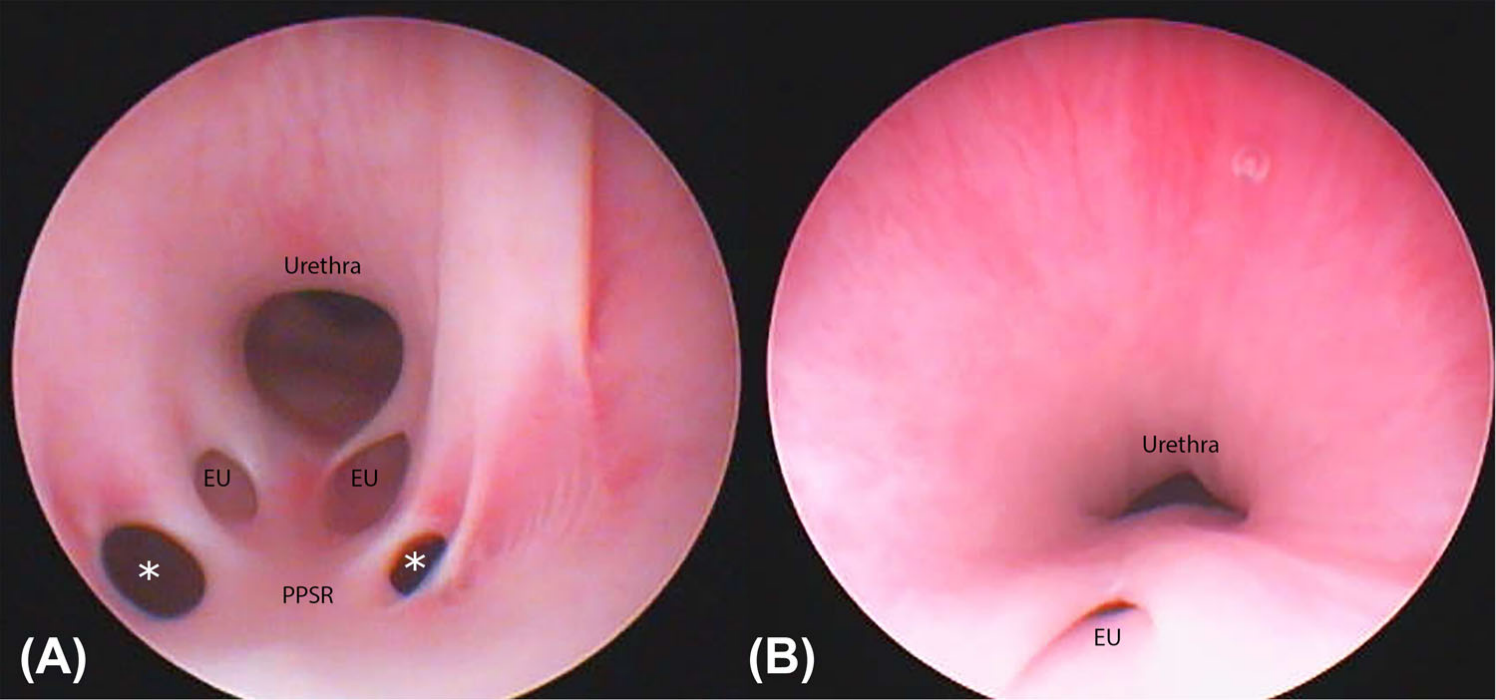
Two still images taken during cystoscopic examination of female dogs. A, A still image showing bilateral ectopic intramural ureteral orifices emptying in the vestibule of a female dog. A persistent paramesonephric septal remnant (PPSR) can also be observed causing stenosis to the vaginal vestibule (demarcated by white asterisks). B, A still image showing a unilateral right‐sided intramural ectopic ureter emptying into the mid‐urethra of a female dog. Both images were acquired with the patients anesthetized in dorsal recumbency using a 2.7 mm HOPKINS Forward‐Oblique Telescope 30° in combination with a 3.5 mm Examination and Protection Sheath (Karl‐Storz Endoscopy Ltd., Slough, UK) [Colour figure can be viewed at wileyonlinelibrary.com]

In all dogs, the cystoscopic examination was performed under general anesthesia by one board‐certified small animal soft tissue surgeon (L.O.) using one of two cystoscopes: a 2.7 mm HOPKINS Forward‐Oblique Telescope 30° in combination with a 3.5 mm Examination and Protection Sheath (Karl‐Storz Endoscopy Ltd., Slough, UK) or a 9.5Fr. Operating Telescope (Karl‐Storz Endoscopy Ltd., Slough, UK). For female dogs, a rigid cystoscope was advanced retrograde into the vestibule and urethral orifice aided by distension of the vestibule and urethra with sterile saline (0.9% NaCl). For male dogs, a perineal approach was made to allow for cystoscopic access as described by Berent et al.^17^ Each ureteral opening was identified, and the lower urinary tract was assessed for visible congenital abnormalities (Figure 5). Cystoscopic diagnosis of an EU was based on direct visualization of the ureteral opening distal to the bladder neck. If the ureteral openings were in the correct position, the dog was determined not to have ureteral ectopia. On cystoscopic examination, all ureteral orifices were identified and described as ectopic or non‐ectopic. Forty‐three of 76 (57%) ureters were ectopic and 33 (43%) ureters were non‐ectopic. All ectopic ureters were classified as intramural using morphology observed by cystoscopy. Sixteen of 43 (37%) intramural EUs had openings located in the proximal urethra, 11 (26%) in the mid‐urethra, 10 (23%) in the distal urethra, and two (5%) within the prostate. The precise location of four (9%) ectopic ureteral openings was not recorded at the time of cystoscopic examination and no recordings were available for retrospective assessment.

Twelve of 38 dogs (32%) were diagnosed with bilateral intramural EUs on cystoscopic examination, 19 of 38 dogs (50%) had unilateral intramural ureteral ectopia and seven of 38 dogs (18%) had both ureters identified as non‐ectopic. Thirty‐one of 38 dogs (82%) were diagnosed with intramural ureteral ectopia (unilateral or bilateral) based on cystoscopic examination. The only ureter suspected to be extramural ectopic based on AUS was identified as non‐ectopic at cystoscopy. Three out of four ureters that were unable to be identified on ultrasonographic examination were non‐ectopic on cystoscopic examination. The remaining ureter that could not be identified on AUS was intramural ectopic, with the ureteral orifice located in the mid‐urethra.

Using cystoscopic examination as the gold standard, for ureters observed on ultrasound, AUS examination had a sensitivity of 87.8% (95% confidence interval, 74.5–94.7%) and specificity of 86.2% (95% confidence interval, 69.4–94.5%) for identification of individual ureters as ectopic or non‐ectopic. AUS examination had a sensitivity of 85.7% (95% confidence interval, 68.5–94.3%) when identifying the left‐sided ureteric ectopia and a sensitivity of 93.8% (95% confidence interval, 71.7–98.9%) with right‐sided ureteric ectopia. When determining if the dog was positive or negative for ureteral ectopia overall, AUS had a sensitivity of 93.6% (95% confidence interval, 79.3–98.2%) and specificity of 100% (95% confidence interval, 64.6–100%).

Of the 40 ureters identified as ectopic on AUS, 36 of 40 were confirmed as ectopic on cystoscopic examination. Of the 30 ureters identified as non‐ectopic on AUS, 25 of 30 ureters were confirmed as non‐ectopic on cystoscopic examination. The diagnostic accuracy of AUS for determining whether an individual ureter was ectopic or non‐ectopic was 80.3% when all 76 ureters, including those not observed on ultrasound, were considered. All four of the ureters incorrectly diagnosed by AUS as ectopic were associated with the right kidney. All ureters identified as ectopic on cystoscopic examination were confirmed as intramural by the cystoscope operator and laser ablation was performed at the point of diagnosis.

Twenty‐nine of 29 dogs identified as positive for ureteral ectopia using AUS were positive on cystoscopy. Seven of nine dogs that were suspected not to have EUs based on AUS were also negative on cystoscopy. The diagnostic accuracy of AUS for determining whether a dog was positive or negative for ureteral ectopia was 94.7%.

The presence of one or more concurrent urinary tract abnormalities (CUTA) was observed during ultrasonographic examination in 92% (35/38 dogs) of the study population and in 100% of dogs with ureteral ectopia (see Table 1).

Dogs diagnosed with ureteral ectopia had a mean total of 3 (mean 2.39 ±1.17) CUTA while dogs that were unaffected by EUs had a mean total of 1 (mean 0.86 ±0.90) CUTA. Dogs with ureteral ectopia had a significantly greater frequency of CUTA than dogs without EUs (*P* = 0.004). Using a cut‐off of 3 or more CUTA had a sensitivity of 48.4% and a specificity of 100% for the diagnosis of EUs in each dog.

EUs were associated with a significantly higher number of Concurrent Ipsilateral Upper Urinary Tract Abnormalities (CIUTA) (2, range 0–4) than non‐EUs (0, range 0–2), (*P* < 0.001; Table 2). Using a cut‐off of two or more CIUTA had a sensitivity of 54.8% and a specificity of 96.9% for the identification of an individual ureter as being ectopic.

Ureteral dilation was observed in 60% (25/42) of ureters that were confirmed as ectopic by cystoscopy, compared with 3% (1/32) of non‐EUs. The two ureters (one dog) that were not clearly identified as either associated with the left or right kidney were excluded from the CIUTA statistical analysis.

## 4 DISCUSSION

This study aimed to evaluate the sensitivity and specificity of AUS for the diagnosis of ureteral ectopia in dogs presenting to a referral institution with urinary incontinence and to assess whether any other AUS findings could be used to aid the diagnosis of ureteral ectopia. A sensitivity of 87.8% was observed when AUS was utilized to identify the presence of an EU when assessing individual ureters, which increased to 93.5% when utilized to determine the presence of ureteral ectopia for each dog, leading us to accept our hypothesis. This is similar to the previously reported sensitivity of 91% for AUS diagnosis of ureteral ectopia when compared to contrast radiography.^14^ The AUS sensitivity reported in this study is similar to that of CT, lower than that of 4D‐CTEU, and greater than that of intravenous urography.^10^, ^12^ The specificity of AUS for EU has not been previously reported. AUS has the benefit that it allows the thorough evaluation of the abdominal viscera, urinary tract, and concurrent abnormalities associated with ureteral ectopia without the requirement for a general anesthetic/heavy sedation or the administration of intravenous contrast agents.

Cystoscopic examination findings were used as the gold standard for comparison with AUS in this study, due to the previously reported 100% sensitivity of this technique.^8^, ^10^ Lamb et al previously compared AUS findings with intravenous urography^14^ but cystoscopy is significantly more sensitive and therefore more suitable as a gold standard comparison. Cystoscopy allows the operator to identify the presence of concomitant lower urogenital tract abnormalities such as persistent vestibulovaginal remnants and to perform a thorough examination of the vagina, vestibule, and cervix in female dogs. It also enables definitive treatment of intramural EUs with laser ablation at the time of diagnosis, which is not possible with modalities such as contrast‐enhanced CT. While cystoscopy has a 100% sensitivity, it cannot be used to evaluate the upper urinary tract, so AUS, CT, or excretory urography must be performed in conjunction for a complete assessment. Cystoscopy in male dogs is also a more technically demanding procedure due to the requirement for use of a very small diameter flexible ureteroscope or for a perineal approach for the introduction of a rigid cystoscope, as well as the presence of prostatic ducts which can appear similar to a ureteral orifice to less experienced operators.^15^ For male dogs, it is therefore useful to have a diagnostic technique such as AUS or CT intravenous urography that can provide a high index of suspicion for the presence of ureteral ectopia and the status of each individual ureter prior to performing cystoscopy.

Previous studies have suggested that female dogs are more likely to present with EUs, which is consistent with the findings reported here, as 97% (30/31) of the dogs diagnosed with EUs were female. Of the dogs that were diagnosed with ureteral ectopia, 71% (22/31) were Golden retrievers and 10% (3/31) were Labradors. The over‐representation of Golden retrievers is not unexpected as the literature suggests they are a predisposed breed,^18^, ^19^ however, the population of dogs investigated for urinary incontinence at the QVSH may also be skewed due to other ongoing research involving Golden retrievers. A total of 12 dogs (32%) were diagnosed with bilateral EUs which is consistent with the previously reported prevalence of 32–64% in EU populations.^11^, ^17^, ^19^ Extramural EUs are reported to be rare in dogs^18^ and all 43 EUs diagnosed in this study were intramural, with the only suspected extramural ectopic ureter identified to be non‐ectopic during the cystoscopic examination. Cystoscopic classification of ectopic ureters as either intramural or extramural has not been described in the literature and classification was reliant on the operator's assessment of the ureter's morphological features. All ectopic ureters were subsequently successfully laser‐ablated confirming the suspicion of an intramural phenotype.

Ultrasound diagnosis of EUs involves the identification of a ureteral jet by doppler, the identification of the tubular ureteral structure distal to the bladder neck, and the presence of secondary hydroureter.^14^ Intravenous administration of furosemide (0.5 mg/kg) has been reported to aid the location of the ureteral orifice in “normal” dogs.^20^ Medical records indicate that at least 1 dog in this study received intravenous furosemide, although the number of dogs that received diuretic is difficult to ascertain as it was inconsistently documented. It may be useful for future studies to assess whether the administration of intravenous furosemide affects the diagnostic accuracy of AUS for identification of EUs.

No extramural EUs were diagnosed by cystoscopy in this study, so the sensitivity and specificity of AUS for the identification of extramural EU was not assessed.

There are limitations regarding the use of AUS as the primary diagnostic imaging modality to diagnose and identify EUs in dogs. The most significant is that AUS is a highly operator‐dependent modality and there is a high level of skill required to consistently acquire diagnostic quality images of a mobile, soft tissue tract such as a ureter. Image acquisition is made more complicated by the presence of other congenital abnormalities, such as a short urethra, lack of a distinct bladder neck, or an intrapelvic bladder.^18^ These abnormalities can make it difficult to perform complete urethral evaluation due to the acoustic shadowing associated with the pelvis. In this study, AUS incorrectly identified one normal ureter as an extramural ectopic ureter. The AUS report described a tubular structure running parallel to the bladder neck and proximal urethra, leading to the assumption that the ureter was extramural. An explanation for this misdiagnosis could be that a blood vessel may have been mis‐identified as a ureter. Even once a ureter is located, there can be difficulties in identifying which ureter is being assessed as the image acquisition in AUS is continuous. One of the dogs in this population was correctly diagnosed with unilateral EU, but the operator was unable to determine which kidney the ureter was associated with. In this case, the lack of identification of the affected side did not ultimately change the treatment plan.

Ureteral ectopia is often associated with the presence of CUTA including hydroureter, an intrapelvic bladder, and hydronephrosis^18^ which can aid in the diagnosis of EU when utilizing AUS. In the population assessed within this study, 100% of dogs with EU had CUTA with the most common being renal pelvis dilation (23/31 dogs) and ureteral dilation/hydroureter (21/31). It is theorized that the presence of these abnormalities is related to the intramural section of the distal ureter creating back pressure of urine leading to altered peristalsis.^18^ This study found that dogs with EUs have a significantly (*P* = 0.004) greater frequency of CUTA than dogs without EUs. If a urinary incontinent dog exhibited ≥3 CUTA on AUS, the results from this study suggest that there is a strong likelihood that the dog has ureteral ectopia. Although this technique is not sensitive enough to be relied upon as a screening technique, it is arguably a useful adjunctive tool to help confirm a positive diagnosis in cases where the ureteral orifice is difficult to locate.

While the ability to identify ectopia accurately in individual ureters is subjectively less important for a screening test, determining whether a dog has CIUTA on the ipsilateral side to the EU may affect the treatment plan. This study found that EUs have a significantly (*P* < 0.001) greater frequency of CIUTA than non‐EUs. If a ureter is associated with ≥2 CIUTA, there is a high likelihood that the ureter is ectopic. Ureteral dilation was the most commonly identified CIUTA associated with an individual EU. 59.5% of EUs exhibited ureteral dilation compared with 3% of non‐EU. The presence of ureteral dilation in conjunction with the identification of ≥2 CIUTA is therefore highly suggestive of an EU. This may be useful to increase the index of suspicion for an EU, even when the location of the ureteral orifice cannot be identified.

Limitations of this study include its retrospective nature and the relatively small study population, which is inherent to the relative rarity of ureteral ectopia and cases referred for investigation of urinary incontinence. As a referral institution, the cases that present to the QVSH have often undergone preliminary investigations and medical treatment trials which may have ruled out common causes of urinary incontinence other than ureteral ectopia, such as urethral sphincter mechanism incompetence, bacterial cystitis, or cystolithiasis. This results in a higher prevalence of ureteral ectopia in the study population than the population presenting to the general practitioner.

As an imaging modality, AUS is highly operator dependent and features such as corticomedullary distinction are highly subjective, which means that the results presented here cannot necessarily be extrapolated to other institutions. Due to the retrospective nature of the study, the surgeon who operated the cystoscopy was not blinded to the findings of the AUS. There is a level of subjectivity when identifying ectopic ureters in dogs with a poorly defined bladder neck and the lack of blinding could have resulted in a potential bias when determining whether a ureter with an opening at the distal aspect of the bladder neck is ectopic. A prospective study with the appropriate blinding is recommended to confirm the findings of this study. In conclusion, AUS is a specific and sensitive initial screening tool for EU diagnosis in dogs with urinary incontinence. In cases where the cost, speed, and risk of the diagnostic technique is a concern, AUS can be used as an alternative to CT as a screening tool and can achieve a higher sensitivity to intravenous excretory urography for identifying intramural EU in dogs. If the ureteral orifice cannot be identified clearly on AUS, diagnosis, and identification of EU in urinary incontinent dogs may be aided by assessing the presence of CUTA and number of CIUTA but further investigation is necessary to characterize this relationship fully.

## 5 LIST OF AUTHOR CONTRIBUTIONS

### Category 1

(a) Conception and Design: Taylor, Knight, Owen
  (b) Acquisition of Data: Genain
  (c) Analysis and Interpretation of Data: Taylor, Knight, Owen

### Category 2

(a) Drafting the Article: Taylor, Knight, Genain, Owen
  (b) Revising Article for Intellectual Content: Genain, Owen

### Category 3

(a) Final Approval of the Completed Article: Taylor, Knight, Genain, Owen

### Category 4

(a) Agreement to be accountable for all aspects of the work in ensuring that questions related to the accuracy or integrity of any part of the work are appropriately investigated and resolved:  Taylor, Knight, Genain, Owen

## CONFLICT OF INTEREST

The authors have declared no conflict of interest.

## PREVIOUS PRESENTATION OR PUBLICATION DISCLOSURE

Presentation of abstract to BSAVA Virtual Congress: The Abstract Sessions 2021.

## EQUATOR NETWORK DISCLOSURE

No EQUATOR network checklist was used.
